# Neural and Psychological Predictors of Cognitive Enhancement and Impairment from Neurostimulation

**DOI:** 10.1002/advs.201902863

**Published:** 2020-01-21

**Authors:** Li‐Zhuang Yang, Wei Zhang, Wenjuan Wang, Zhiyu Yang, Hongzhi Wang, Zhi‐De Deng, Chuanfu Li, Bensheng Qiu, Da‐Ren Zhang, Roi Cohen Kadosh, Hai Li, Xiaochu Zhang

**Affiliations:** ^1^ Anhui Province Key Laboratory of Medical Physics and Technology Center of Medical Physics and Technology Hefei Institutes of Physical Science Chinese Academy of Sciences Hefei Anhui 230031 China; ^2^ Cancer Hospital Chinese Academy of Science Hefei Anhui 230031 China; ^3^ Hefei National Laboratory for Physical Sciences at the Microscale, and School of Life Sciences University of Science and Technology of China Hefei Anhui 230027 China; ^4^ Noninvasive Neuromodulation Unit Experimental Therapeutics & Pathophysiology Branch Intramural Research Program National Institute of Mental Health National Institutes of Health Bethesda MD 20892‐9663 USA; ^5^ Laboratory of Digital Medical Imaging Medical Imaging Center First Affiliated Hospital Anhui University of Chinese Medicine Hefei Anhui 230031 China; ^6^ Center for Biomedical Engineering University of Science and Technology of China Hefei Anhui 230027 China; ^7^ Department of Experimental Psychology University of Oxford Oxford OX1 3UD UK; ^8^ Academy of Psychology and Behavior Tianjin Normal University Tianjin 300387 China; ^9^ Hefei Medical Research Center on Alcohol Addiction Anhui Mental Health Center Hefei 230022 China

**Keywords:** lateralization, medial superior frontal gyrus, neurostimulation, tDCS, temporoparietal junction

## Abstract

Modulating the temporoparietal junction (TPJ), especially the right counterpart, shows promises in enhancing social cognitive ability. However, it is ambiguous whether the functional lateralization of TPJ determines people's responsiveness to brain stimulation. Here, this issue is investigated with an individual difference approach. Forty‐five participants attended three sessions of transcranial direct current stimulation (tDCS) experiments and one neuroimaging session. The results support the symmetric mechanism of left and right TPJ stimulation. First, the left and right TPJ stimulation effect are comparable in the group‐level analysis. Second, the individual‐level analysis reveals that a less right‐lateralized TPJ is associated with a higher level of responsiveness. Participants could be classified into positive responders showing cognitive enhancement and negative responders showing cognitive impairment due to stimulation. The positive responders show weaker connectivity between bilateral TPJ and the medial prefrontal cortex, which mediates the prediction of offline responsiveness by the lateralization and the social‐related trait. These findings call for a better characterization and predictive models for whom tDCS should be used for, and highlight the necessity and feasibility of prestimulation screening.

## Graphical abstract

People can either be positive or negative responders to brain stimulation. A less degree of functional asymmetry of the temporoparietal junction (TPJ) and a higher degree of social‐related difficulties predicts more beneficial effects for both left and right TPJ stimulation. The medial prefrontal cortex, which couples with bilateral TPJ, is a potential hub modulating the brain stimulation effect.

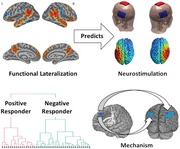

## 1 Introduction

Neurostimulation techniques, particularly transcranial direct current stimulation (tDCS), have become very popular due to their potential clinical value, low‐cost and minimal side effects. Although tDCS is easy to operate, it is not so easy to achieve consistent outcomes, as the effect size and even the direction of the stimulation effect varies markedly across individuals.^1^

Typical tDCS montages often imply lateralized stimulation because of the ipsilateral electrode configuration or the effect of current polarity in the symmetrical electrode configuration.^2^ For example, tDCS had been used in stroke rehabilitation by inhibiting the unaffected hemisphere or facilitating the excitability of the affected hemisphere according to the interhemispheric competition model of the stroke.^3^ However, the degree to which asymmetric interhemispheric inhibition impacts on stroke recovery is controversial.^4^, ^5^ There seems to be an appealing and intuitive link between functional lateralization and stimulation effect. However, the nature and direction of this link are poorly understood, especially in the high‐level cognition, such as social cognitive processing.

We study the association between functional lateralization and neurostimulation responsiveness by focusing on the temporoparietal junction (TPJ),^6^, ^7^, ^8^ which has received considerable interest in recent years as its nexus role in attention, memory, language, and social cognition.^9^ TPJ, especially the right TPJ, is a vital hub of the social brain.^10^ One underlying computational mechanism of TPJ might be controlling the self and other representation flexibly according to the context and goal.^11^ For example, people should differentiate other's belief from self's belief (theory‐of‐mind, ToM),^12^ suppress self's visual perspective when taking other's visual perspective (perspective taking),^13^ and inhibit other's motor representation to perform goal‐directed action (imitation inhibition).^14^, ^15^ Emerging evidence suggests a functional‐anatomical overlap of ToM, self‐other distinction, and imitation inhibition in TPJ.^16^, ^17^ The dysfunction of TPJ accompanies with several psychiatric diseases characterized by social deficits, such as schizophrenia and autism.^18^, ^19^, ^20^ Noninvasive brain stimulation over TPJ can modulate participants' social cognitive abilities,^11^, ^21^, ^22^, ^23^, ^24^ suggesting TPJ as a promising stimulation target for social cognitive enhancement.

There is evidence that the involvement of TPJ in social cognition is right‐lateralized.^25^, ^26^, ^27^, ^28^ Despite this, the function of the left TPJ is less understood, and its role in social cognition might be overlooked.^29^ According to a recent meta‐analysis, both the left and right TPJ were shown to be involved in ToM tasks, albeit with stronger activation in the right TPJ.^10^ Lesion studies also supported the necessity of left TPJ for representing other's belief.^30^, ^31^ A recent tDCS study also found that the left and right TPJ stimulation effect on social cognitive abilities are comparable.^14^ However, the group‐level analysis might neglect the heterogeneity among participants. It is unclear whether individual TPJ lateralization could modulate the tDCS effect. Besides, the direction of the association is also ambiguous. For example, do those with a stronger degree of right lateralization benefit more from a right‐side stimulation than a left‐side stimulation? The current knowledge could not give us a reasonable answer.

**Figure 1:**
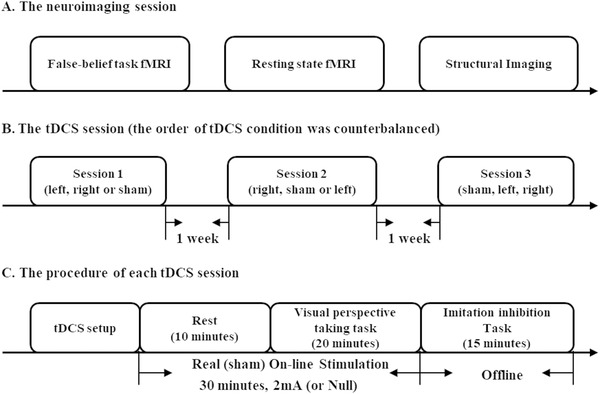
The design of the study. A) Participants first attended a neuroimaging session, including a false‐belief fMRI localizer task, a resting‐state fMRI, and *T*_1_‐weighted structural imaging. B) Participants completed three tDCS sessions of different conditions (left TPJ stimulation, right TPJ stimulation, and sham stimulation). The order of the tDCS condition was counterbalanced and separated by one week. C) In each tDCS session, participants were first equipped with tDCS. Then, they received a kind of tDCS stimulation for 30 min. The assignment of the tDCS condition was blind to participants. During the left or right stimulation, the current ramped up over 30 s, maintained a current of 2 mA for 29 min, and ramped down over 30 s. During sham stimulation, the current started and ended within 1 min in a ramp‐like fashion. In the first 10 min of stimulation, participants were instructed to be calm down and have a rest. To prevent them from noticing the possible tingling effect at the initial stage of electrical stimulation, they were allowed to play a mental rotation task on the screen with the explicit instruction that this task was just for recreation, and their performance on this task was not recorded and analyzed. Then they completed a visual perspective‐taking task for 20 min. After the electricity terminated, they completed an imitation inhibition task for 15 min. Participants were instructed to respond as quickly as possible with the accuracy emphasized.

The present study addressed this issue with an individual difference approach. Participants in our study attended three sessions of tDCS experiments and one neuroimaging session (**Figure** **1**). In the tDCS experiment session, behavioral measures during (online) and immediately after (offline) stimulation in left, right, and sham condition was collected. The within‐subject design of tDCS experiment in the present study enables a fair comparison between the left and the right TPJ stimulation effect at the group‐level analysis. Moreover, the design of the study enables us to examine the link between the functional asymmetry of TPJ and neurostimulation responsiveness at the individual‐level analysis.

**Figure 2:**
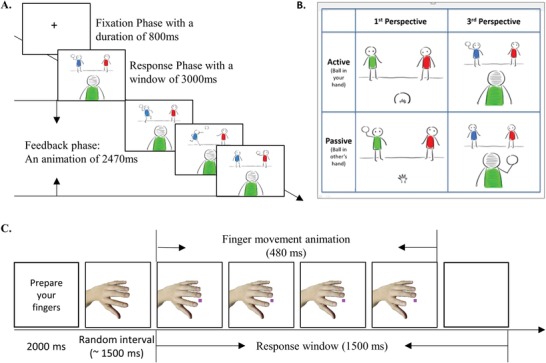
The behavioral task used during the online and offline tDCS phase. A) An illustration of a trial in the active mode and third‐person perspective. The participant served as the agent of the blue avatar, who need to throw the ball to the red avatar by a button press on a keyboard within 3 s. A feedback movie (2.47 s) was presented according to the participant's response. B) The design of the visual perspective‐taking task. The participant's visual perspective was either the same (the first‐person perspective, 1PP) or different (the third‐person perspective, 3PP) with the perspective of the blue avatar. In one‐third of the trials (active condition), the participant threw the ball—by a button press on a keyboard—to the red avatar by indicating whether the red avatar was on the left or the right side of the blue avatar. In remaining trials (passive condition), the participant indicated whether the avatar on his (or her) left or right side with the ball in hand. C) An illustration of a typical incongruent trial in the imitation inhibition task. The participant had to lift his/her middle finger on the keyboard when the square was purple. Simultaneously, a hand on the screen lifted the index finger. The participant had to inhibit the representation of an incongruent motion by others and lift the middle finger accordingly in this trial.

For online stimulation, we used a visual perspective‐taking task,^13^ targeting taking other's visual perspective by inhibiting self‐representation (**Figure** **2**A,B). The view switching cost, measured as the relative cost in the third‐person perspective over the first‐person perspective, was chosen as the behavior index of visual perspective‐taking ability. We defined the online responsiveness as the relative view switching cost in the sham condition over the real stimulation condition, with a positive value indicating an enhancement of perspective‐taking performance by stimulation. To measure offline behavioral performance, we used the imitation inhibition task,^14^, ^22^ which target the ability to inhibit other's task‐irrelevant motor representation (Figure 2C,D). The imitation cost, measured as the relative cost in the incongruent finger movement condition over the congruent finger movement condition, was used as the behavior index of imitation inhibition. We defined the offline responsiveness as the relative imitation cost in the sham condition over the real stimulation condition, with a positive value indicating an enhanced ability in stimulation condition to resist the automatic imitation tendency.

**Figure 3:**
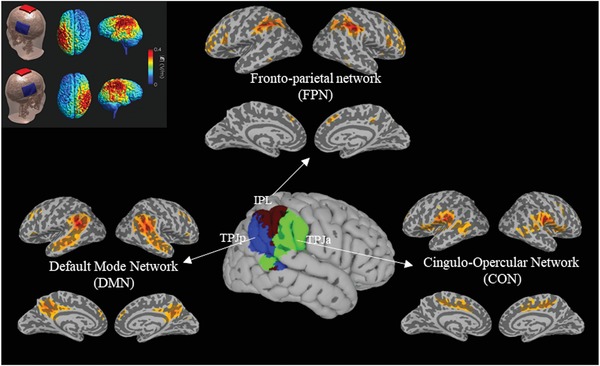
The functional division of TPJ (middle) and the modeling of electrical distribution (the top‐left corner). The network maps were the mean fisher‐z transformed correlation map using the sample of the study (*n* = 45). An arbitrary threshold of *z* > .30 and a cluster size of 30 voxels was chosen for visual illustration. The electric field distribution was simulated using SimNIBS 2.1 (https://simnibs.de/) in a standard brain. *T*_1_‐ and *T*_2_‐weighted MRI was segmented into scalp, skull, cerebrospinal fluid, gray matter, and white matter. Furthermore, two 5 cm × 7 cm sponge electrodes pads were placed on CP5 and CP6 according to the International 10–20 EEG system. The electric field was solved using the finite‐element method with an input current of 2 mA.

We captured the functional property of TPJ in social cognition using the false‐belief fMRI localizer task, a well‐validated tool to locate ToM processing. We quantified the functional asymmetry of TPJ using the lateralization index (LI) with a bootstrap approach,^32^ with a positive value indicating rightward lateralization. As the heterogeneity of the broadly defined TPJ,^8^, ^33^, ^34^ we divided the TPJ into IPL, TPJa and TPJp from a network view^7^, ^8^, ^35^ (**Figure** **3**) and examined their separate contribution in predicting neurostimulation responsiveness. To investigate whether intrinsic neural traits might mediate the association between the LI and neurostimulation responsiveness, we also performed a functional connectivity analysis with the available individual resting‐state fMRI data.

Personality traits might be an indispensable modulating variable in the tDCS research. For example, tDCS over dorsal lateral prefrontal cortex improved arithmetic performance in individuals with high anxiety but not for people with low mathematical anxiety.^36^ In case of TPJ stimulation, Donaldson and colleges demonstrate that autism‐relevant characteristics interact with the stimulation effect on social cognition,^37^ suggesting that the social trait might be a modulating variable of TPJ stimulation effect. Thus, in addition to the brain measures, we also collected two psychological indexes on social‐related personality traits (social trait), such as the degree of social phobia^38^ and autism.^39^

## 2 Results

### 2.1 The Lateralization of TPJ

**Figure 4:**
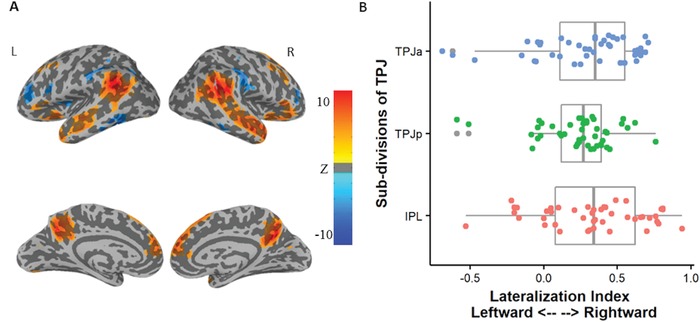
The functional activation of false‐belief reasoning and the distribution of the lateralization index. A) Group‐wise whole‐brain analysis of the belief‐photo contrast with a voxel‐wise *p* threshold of .001 and cluster‐wise threshold of .01. B) The boxplot of LI (gray) in each sub‐division of TPJ with jittered LI values (colored points) added. The minimum, first quartile, median, third quartile, and the maximum was displayed using the whisker and box.

The whole brain analysis of the false‐belief task yielded a typical theory‐of‐mind network, composing of midline brain structures (such as medial frontal gyrus and posterior cingulate cortex) and bilateral temporal parietal regions (**Figure** **4**A). All three TPJ divisions showed a rightward lateralization in general manifested by the lateralization index [LI_IPL: *M* = .338, *SD* = .343, *t* = 6.600, *df* = 44, *p* < .001; LI_TPJa: *M* = .292, *SD* = .346, *t* = 5.665, *df* = 44, *p* < .001; LI_TPJp: *M* = .236, *SD* = .257, *t* = 6.177, *df* = 44, *p* < .001] (Figure 4B). The LI indices in the three TPJ divisions were comparable [*F* (2, 88) = 1.687, *p* = .191, *η^2^* = .017].

### 2.2 The tDCS Effect at the Online and Offline Phase

**Figure 5:**
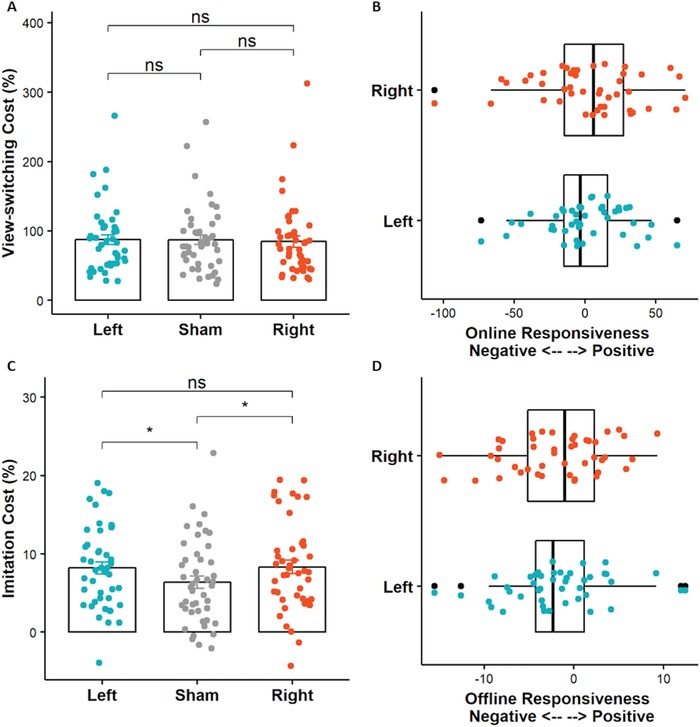
The online and offline tDCS effect and individual neurostimulation responsiveness. A) The online tDCS effect. B) The individual online left and right neurostimulation responsiveness. C) The offline tDCS effect. D) The individual offline left and right neurostimulation responsiveness. * indicates *p* < .05.

Both left and right stimulation failed to modulate the view‐switching cost at the group level during the online phase [*F*(2, 88) = .260, *p* = .772, *η^2^* = .0007] (**Figure** **5**A). The individual online neurostimulation responsiveness for left and right stimulation was illustrated by Figure 5B. See also Tables S1, S2, and S3 in the Supporting Information for summary of omnibus ANOVA for raw behavior measures.

A significant offline tDCS effect on imitation cost at the offline phase was observed [*F*(2, 88) = 3.554, *p* = .033, *η^2^* = .027] (Figure 5C). Both the left stimulation and right stimulation increase the imitation cost compared with the sham stimulation [left: *t* = 2.177, *df* = 44, *p* = .035, Cohen's *d* = .325; right: *t* = 2.384, *df* = 44, *p* = .022, Cohen's *d* = .355], suggesting an enhancement induced by tDCS. Moreover, the left and right stimulation effect was comparable [*t* = .179, *df* = 44, *p* = .859, Cohen's *d* = .027] (Figure 5D). See also supporting information for complementary analysis on raw measures such as response time and accuracy (Tables S4, S5, and S6, Supporting Information).

### 2.3 The Association between LI and Neurostimulation Responsiveness

**Figure 6:**
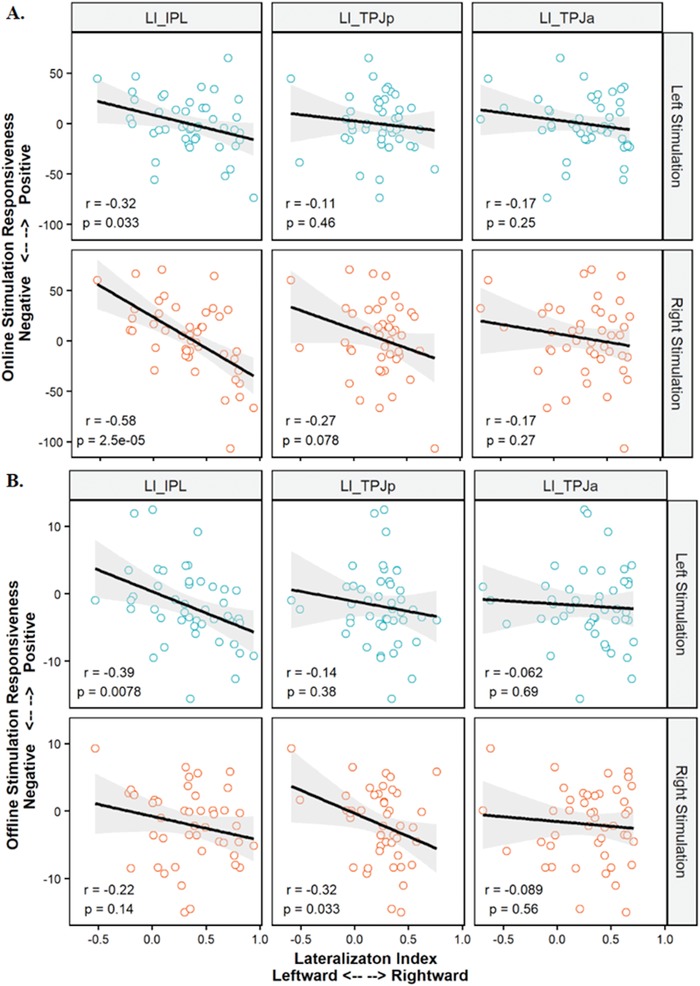
The association between the neurostimulation responsiveness and lateralization index. A) Online neurostimulation responsiveness predicted by the lateralization index of IPL, TPJp, and TPJa. B) Offline neurostimulation responsiveness predicted by the lateralization index of IPL, TPJp, and TPJa.

For online stimulation, we found a general negative association between neurostimulation responsiveness and LI for both left and right stimulation (**Figure** **6**A). The LI of IPL predicted the left and the right neurostimulation responsiveness [left: *r* = –.319, *p* = .033; right: *r* = –.584, *p* < .001]. For offline stimulation, we also found a consistent negative association between the neurostimulation responsiveness and the LI of IPL [left: *r* = –.392, *p* = .008; right: *r* = –.221, *p* = .143] (Figure 6B).

To compare the contribution of LI of the three sub‐divisions of TPJ, we fitted 4 regression models with the three LIs as predicting variables to explain online left neurostimulation responsiveness, online right neurostimulation responsiveness, offline left neurostimulation responsiveness and offline right neurostimulation responsiveness, respectively (See Tables S7, S8, S9, and S10 in the Supporting Information for detail statistical summary for each regression model). The standard regression coefficients were calculated. We found a consistent contribution of the LI of the IPL after controlling the contribution from LI of TPJa and TPJp (see Figure S1 in the Supporting Information for an illustration), for online left [β = –.345, *SE* = .158, *t* = –2.181, *p* = .035], online right [β = –.575, *SE* = .124, *t* = –4.205, *p* < .001], and offline left [β = –.400, *SE* = .156, *t* = –2.563, *p* = .014] neurostimulation responsiveness.

To examine the specific contribution of left and right TPJ, especially the IPL, in predicting the neurostimulation responsiveness, we fitted 4 regression models with left and right activations of IPL as predicting variables to explain the online left neurostimulation responsiveness, the online right neurostimulation responsiveness, the offline left neurostimulation responsiveness, and the offline right neurostimulation responsiveness, respectively. Intriguingly, we found a consistently significant positive contribution of left IPL activations and negative contribution of right IPL activations in predicting the online right neurostimulation responsiveness (see Tables S11, S12, S13, and S14 in the Supporting Information).

### 2.4 The Positive Responders and the Negative Responders

#### 2.4.1 Hierarchical Cluster Analysis

**Figure 7:**
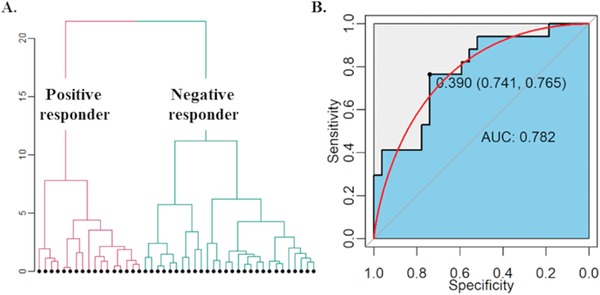
Responder classification and prediction. A) Classifying participants into positive responders and negative responders according to their online and offline responsiveness profile. B) Predicting responder type using lateralization index and social‐related trait.

A hierarchical cluster analysis was performed to classify subjects according to their tDCS response profile, namely the online left, online right, offline left, and offline right neurostimulation responsiveness. The cluster analysis revealed coarsely two groups: the positive responders and the negative responders (**Figure** **7**A). The positive responders showed significant positive neurostimulation responsiveness indicating a gain of performance after active stimulation, while the negative responders showed significant negative neurostimulation responsiveness indicating a loss of performance after active stimulation (see Figure S2 in the Supporting Information for an illustration).

#### 2.4.2 Profile of the Positive Responder

The positive responders had a lower level of rightward lateralization of IPL than the negative responders [*t* = 2.953, *df* = 30.211, *p* = .006, Cohen's *d* = .941] (Figure S3, Supporting Information). However, there was no difference between the positive responders and the negative responders on lateralization of TPJa [*t* = .071, *df* = 26.938, *p* = .944, Cohen's *d* = .023] and TPJp [*t* = 1.388, *df* = 35.966, *p* = .174, Cohen's *d* = .419]. Moreover, the positive responders showed a stronger left IPL activation than the negative responders [*t* = 2.314, *df* = 30.027, *p* = .028, Cohen's *d* = .739]. However, the two group were not different from each other on the right IPL activation [*t* = .005, *df* = 34.718, *p* = .996, Cohen's *d* = .002] (Figure S4, Supporting Information). Interestingly, the positive responders and the negative responders also differed on social traits. Specifically, positive responders scored higher on the score of the social phobia inventory (Table S15, Supporting Information).

Utilizing the composite LI (mean of the LI of TPJa, TPJp and IPL) and the composite social trait score (mean of the Autism Quotient and social phobia scale), we can train a model to identify the positive responders successfully. A systematic model comparison procedure verified that the LI and the social‐related trait had unique and independent contributions (See supporting information for detailed information). We also evaluated the performance of those three models using leave‐one‐out‐cross‐validation (LOOCV), which picked the model without interaction term as the most optimal (see Table S16 in the Supporting Information for detail information). The Receiver Operating Curve analysis on the optimal model suggests a good sensitivity and specificity in the classification performance (area under the curve: 0.782) (Figure 7B). The LI and the social trait all have significant contributions (LI: β = –4.931, *SE* = 2.410, *Z* = –2.046, *p* = .041; Social Traits: β = .118, *SE* = .047, *Z* = 2.518, *p* = .012). The leave‐one‐out‐cross‐validation also verified a reasonable generalizability (Accuracy = .636, Kappa = .198).

### 2.5 The Mechanism under the Association between LI and Neurostimulation Responsiveness

**Figure 8:**
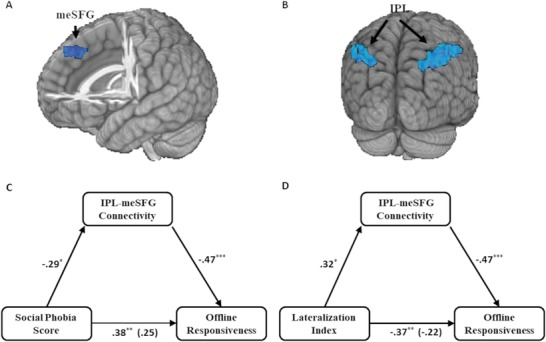
The IPL‐meSFG coupling and its mediating role. A) Whole‐brain connectivity analysis using the mean time‐series of bilateral IPL part of TPJ as seed demonstrated that the positive responders showed weaker connectivity in the medial superior frontal gyrus (meSFG) than negative responders [Peak MNI coordinate: *x* = 1, *y* = 34, *z* = 46, voxel‐wise *p* threshold = .001, cluster size = 1431 mm^3^, cluster‐wise *p* < .04, determined by the ‐3dClustsim option embedded in 3dttest^++^ of AFNI]. B) Further brain‐wide analysis using the meSFG as a seed verified that positive responders have both weaker left IPL‐meSFG connection [Peak MNI coordinate: x = –50, *y* = –44, *z* = 40, voxel‐wise *p* threshold = .001, cluster size = 2187 mm^3^, cluster‐wise *p* < .02] and right IPL‐meSFG connection [Peak MNI coordinate: *x* = 34, *y* = –47, *z* = 40, voxel‐wise *p* threshold = .001, cluster size = 7317 mm^3^, cluster‐wise *p* < .01]. Notice that the analysis using the meSFG as the seed was merely used to illustrate both the left and right IPL showed weakened connectivity in positive responders. The IPL‐meSFG connectivity fully mediated the association between social phobia score and offline responsiveness (C) and the association between lateralization index and offline responsiveness (D). Notice that the number beneath the arrow body indicates the standardized regression coefficients and its significance level. The standardized regression coefficients controlling for the IPL‐meSFG connectivity was shown in parentheses. * indicates *p* < .05; ^**^ indicates *p* < .01; ^***^ indicates *p* < .001.

Our results yielded a comparable left and right TPJ stimulation effect. Also, the individual analysis indicates the positive responders to tDCS showed a less rightward lateralization of TPJ, especially at the IPL part, than the negative responders. These pieces of evidence suggest a similar mechanism might underlie the left and right TPJ stimulation effect. Both left and right TPJ stimulation effect thus might mediated by a third network hub. To examine this hypothesis, we performed a seed‐based analysis using the bilateral IPL as the seed and compared the IPL network strength between positive responders and negative responders using whole‐brain *t*‐test. Our results showed that positive responders showed weaker functional connectivity between IPL and medial superior frontal gyrus (meSFG) than the negative responders [voxel‐wise *p* threshold = .001, cluster size = 1431 mm^3^, cluster‐wise *p* value <.04] (**Figure** **8**A). Further brain‐wide analysis using the meSFG as seed verified that positive responders have weaker left IPL‐meSFG connection [voxel‐wise *p* threshold = .001, cluster size = 2187 mm^3^, cluster‐wise *p* value <.02] and right IPL‐meSFG connection [voxel‐wise *p* threshold = .001, cluster size = 7317 mm^3^, cluster‐wise *p* value <.01] (Figure 8B).

As Figure 8C illustrates, the standardized regression coefficient between social phobia score and offline neurostimulation responsiveness was statistically significant, as was the standardized regression coefficient between the IPL‐meSFG coupling and offline neurostimulation responsiveness. The standardized indirect effect was .14. We tested the significance of this indirect effect using bootstrapping procedures. The 95% confidence interval of the standardized indirect effect ranged from .01 to .33, suggesting a statistically significant indirect effect. The IPL‐meSFG coupling also mediates the association between LI and offline responsiveness (see Figure 8D for an illustration). The standardized indirect effect was −.15, the 95% confidence interval of the standardized indirect effect, generated by bootstrapping, ranged from −.27 to −.03, indicating the indirect effect of the IPL‐meSFG coupling was also statistically significant in mediating the association between LI and offline neurostimulation responsiveness.

## 3 Discussion

The current study systematically investigates the association between functional TPJ lateralization and responsiveness to lateralized TPJ stimulation using an individual difference approach. Although a general rightward asymmetry of TPJ was observed in the false‐belief task, we found no difference between the left and right stimulation in the group level tDCS effect, suggesting the involvement of bilateral TPJ in social processing. Moreover, individual difference analysis revealed that a weak level of right lateralization favored stronger positive neurostimulation responsiveness, even for the right TPJ stimulation. A further analysis using hierarchical cluster analysis identified two groups: the positive responders and the negative responders. The positive responders, who benefited from both left and right stimulation, had a lower level of right lateralization, which was attributed to stronger activation in the left TPJ, and scoring higher on the social phobia scale than the negative responders. Moreover, the positive responders showed weaker functional coupling between the IPL part of TPJ and the medial superior frontal gyrus, which mediated the prediction of offline neurostimulation responsiveness by lateralization index.

### 3.1 The Necessity and Feasibility of Prestimulation Screening

As the rising tide of tDCS in the public media and academic literature, commercial or “Do‐it‐yourself” tDCS services has become popular nowadays.^40^ However, the tDCS is not such an “easy‐to‐use” technique.^1^ One notorious fact is that the tDCS effect are highly variable across different people.^41^ Many individual characteristics modulate the neurostimulation responsiveness, such as the baseline cognitive ability,^36^, ^42^, ^43^ personality traits,^37^ and age.^44^ The identical tDCS setting might even induce the opposite impact for different people. For example, as revealed by our research, tDCS enhanced task performance in some participants while impaired task performance in the others. The scientific community has recognized the emergency to prevent excessive usage of tDCS.^45^ In addition to the appeal in the scientific community, we propose that developing standardized and easy‐to‐use prestimulation screening procedures might help the implantation of the principle to practice.

Easy‐to‐calculate and simple brain measures, such as the lateralization index (LI), thus has great potential in prestimulation screening. The tDCS montage, no matter ipsilateral or bilateral, implies stimulation over a brain region at one side of the brain. Our study clarifies the intuitive link between the degree of functional lateralization and varied responsiveness to brain stimulation. As illustrated in our research, the degree of rightward lateralization of TPJ is negatively associated with neurostimulation responsiveness, even for the right stimulation. The association can generalize from the visual perspective‐taking task at the online phase to the imitation task at the offline phase, which indicates the feasibility of prestimulation screening using task‐based fMRI. Our fMRI research echoes the recent advances in predicting transcranial random noise stimulation effect using electrophysiology technique.^46^ This line of research advocates the value of individual neuroimaging at baseline in prestimulation screening.

Moreover, psychological scales might also help locate those people that may benefit from tDCS. In the case of TPJ stimulation, our results demonstrate that positive responder, who benefits from stimulation, scores higher on social‐related characteristics, such as the social phobia. The finding is consistent with a recent finding that autism‐relevant traits interact with temporoparietal junction stimulation effect on social cognition.^37^ Besides, our results validate a simple logistic model using the LI and the social‐related trait, which can identify positive responders with a reasonably good performance.

### 3.2 The Contribution of IPL in TPJ Stimulation Effect

According to the network‐based perspective, the broadly defined TPJ divides into three components: The IPL related to the FPN network, the TPJa related to the CON network, and the TPJp related to the DMN network.^7^, ^35^ The TPJa is more close to the center of stimulating electrodes (the CP5/6 in terms of the 10–20 international system of EEG). While the TPJp is more frequently associated with social cognition. However, our results reveal a unique contribution of the IPL in predicting responsiveness to tDCS stimulation over TPJ. Specifically, the LI of IPL could predict responses to tDCS stimulation even after regressing out LI of the other two parts of TPJ. Besides, the left IPL and the right IPL exert opposite impacts on neurostimulation responsiveness. More activations in the left IPL but fewer activations in the right counterpart was associated with higher positive responsiveness.

The IPL, especially the left IPL, takes charge of general‐purpose computation such as working memory, which is frequently recruited by language and social cognitive tasks (Bzdok et al., 2016). In contrast, the right TPJ, including the right IPL, might be more related to social representation (Saxe and Wexler, 2005). Therefore, our results might suggest the functional activation of the left IPL in the false‐belief task reflects the cognitive processing to maintain the task: constructing the scene from reading the story and making a logic reasoning, which maybe play important roles in social cognitive function.

### 3.3 Resolving the Paradox of TPJ Lateralization: The mPFC Matters

The unique role of the right TPJ in social cognition has received considerable interest in recent years.^12^, ^28^, ^47^, ^48^ A series of neurostimulation studies have demonstrated the causal association between right TPJ and some specific social cognitive processes, such as moral judgment,^49^ imitation inhibition,^15^ lie detection,^23^ and visual perspective‐taking.^22^ However, the crucial role of left TPJ in social cognition was also supported. For example, lesions to the left TPJ can impair false‐belief reasoning ability.^30^ Two patients with acquired damage to the left posterior TPJ were unable to take into account other's beliefs unless they were explicitly instructed to do so.^31^ A recent tDCS study directly examined the difference between the left and the right TPJ stimulation,^14^ which found comparable left and right stimulation effects on three tasks: imitation inhibition, perspective‐taking, and ToM.^14^ The controversies in the literature comprise the paradox of TPJ lateralization.

Our study can help clarify the TPJ lateralization paradox. Our results cohere with Santiesteban and her colleague's finding of comparable left and right TPJ stimulation effects on social cognition.^14^ Unlike Santiesteban's research, we used a within‐subject design and measured the online‐ and offline neurostimulation responsiveness using two different tasks. Besides, our individual‐level analysis revealed a negative association even between the rightward lateralization of TPJ and responsiveness to the right TPJ stimulation, which do not favor the right TPJ dominance hypothesis.

The comparable role of bilateral TPJ might be explained from a network view. Precisely, the healthy function of bilateral TPJ might depend on functional coupling with the same neurobiological substrate, such as the midline brain structures. In the literature of social cognition, the TPJ supports mental state reasoning while the dorsal mPFC serves as a hub representing the triadic relationship of the representation of “Me,” “Your,” and “This.”^12^ Thus, both the left and right TPJ might need to interact with the dorsal mPFC in social‐related tasks. Stimulating TPJ, no matter the left or right part, shared the same mechanism of influencing the mPFC. This hypothesis is consistent with the recent network explanation of the tDCS effect.^50^, ^51^, ^52^

The mediation analysis framework can enable an examination of the network explanation using an individual difference approach.^53^ We found that the functional coupling between the IPL part of TPJ and meSFG can fully mediate the association between TPJ lateralization and neurostimulation responsiveness. The meSFG we found is a part of dorsal mFPC, which overlaps with the cluster yielded in a previous study (peak MNI coordinate: *x* = –2, *y* = 26, *z* = 44).^54^

### 3.4 Limitations and Future Directions

The promise of TPJ stimulation as a treatment of social deficits has been advocated.^18^, ^19^, ^21^, ^22^ It would be ideal that the degree of social deficits was associated with the neurostimulation responsiveness, which could support the usage of noninvasive brain stimulation in clinical settings. The work of Donaldson and his colleagues manifests the association between autism trait and brain stimulation effect.^37^ Our results provide some further preliminary evidence. First, we demonstrate that positive responders were those with a higher level of social anxiety and autism. Second, the social phobia scale could predict the offline right TPJ stimulation effect. However, participants in our study were healthy young adults. Future studies might investigate the TPJ stimulation responsiveness using a sample of patients characterized by social dysfunction, such as autism and schizophrenia. Besides, the role of other kinds of lateralization, such as handedness and morphology asymmetry, contributes to neurostimulation responsiveness should be clarified in the future with large sample size.

Moreover, although our analysis suggests that the TPJ‐mPFC coupling might be the shared mechanism of both left and right TPJ stimulation, we could not test this hypothesis directly as no neuroimaging data during the tDCS session collected in the present study. Future studies might combine the tDCS and fMRI scanning to examine the shared and distinct neurobiological mechanisms under the left and right TPJ stimulation. Another valuable research question is whether the task during the brain stimulation stage would interact with the brain stimulation effect. Besides, utilizing a baseline performance measurement instead of a sham control might help to clarify the mechanism.

## 4 Conclusion

tDCS is becoming more and more popular in recent years, which might promote excessive usage. Our study demonstrated the heterogeneity in neurostimulation responsiveness, and provided clear biomarkers and cognitive traits that predict enhancement and impairments effects due to the usage of tDCS. Our findings call for a better characterization and predictive models for whom tDCS should be used for, and highlight the role of medial frontal cortex in mediating the TPJ stimulation effect.

## 5 Experimental Section

##### Participants

Participants were recruited from the USTC campus by advertisement. Individual telephone interviews screened the potential participants. Only those with normal or corrected‐to‐normal vision, right hand dominated assessed with the Edinburgh Handedness Inventory,^55^ and without neurologic and psychiatric histories were enrolled. Fifty‐four young adults attended the study. Eight participants were excluded for failing to complete the fMRI session (*n* = 1), or the tDCS session (*n* = 7), or extreme MRI image distortion (*n* = 1).The final sample for data analysis was 45 participants [24 females, age mean (SD) = 22.44 (2.24)]. All participants filled in informed consent forms before study participation and received monetary compensation after the completion of all sessions. The Institutional Review Board of USTC approved the study.

##### Study Visits

Participants attended an MRI scanning session and three tDCS sessions (Figure 1). The MRI session consisted of functional images of the false‐belief task, resting state, and anatomical *T*_1_ images. In the tDCS experiment, participants attended three sessions of stimulation that aimed to target the right TPJ, left TPJ, and sham stimulation (named left stimulation condition, right stimulation condition, and sham stimulation condition in the following text). The order of stimulation was randomized and was blind to participants and the experimenter. The time interval between tDCS sessions was at least 72 h to negate the potential aftereffects of tDCS. During each tDCS session, participants had a rest for 10 min and then completed a visual perspective‐taking task. Immediately after the stimulation terminated, participants completed an imitation inhibition task to measure their ability to inhibit other's motor representation. Participants also completed two psychological scales, the Social Phobia Inventory^38^, ^56^ and the Autism Quotient Scale,^39^, ^57^ usually at the end of their first visit to our lab. One participant did not complete the two scales for personal reasons.

##### The tDCS Protocol

Direct current was applied by two electrodes inserted into saline‐soaked synthetic sponges (5 × 7 cm^2^) and a battery‐driven stimulator (DC‐Stimulator Plus, neuroConn GmbH, Ilmenau, Germany). There were three sessions of tDCS experiment: left stimulation, right stimulation, and sham stimulation. For active stimulation, the center of the anode was placed on the CP5 for the left stimulation condition or the CP6 for the right stimulation condition, according to the electroencephalography (EEG) 10/20 system; the center of the cathode was located at the vertex (Figure 3). For sham stimulation, the configuration was either the same as the right stimulation or the left stimulation, which was randomized in the experiment. The placement of electrodes was aided by an elastic Quick EEG cap (Neuroscan Inc., USA). During active stimulation, the current ramped up over 30 s, maintained a current of 2 mA for 29 min, and ramped down over 30 s. During sham stimulation, the current started and ended within 1 min in a ramp‐like fashion. The order of stimulation was randomized and was blind to participants and the experimenter. Participants were required to wash and dry their hair carefully in our lab before setting up the tDCS montage to reduce the skin resistance to 5 kΩ, even in the sham condition. A mini‐tDCS test was used to check the skin resistance level.

##### Behavior Tasks and Definition of Neurostimulation Responsiveness: Behavior Measure During Online Stimulation—

The visual perspective‐taking was measured using the ball‐tossing task.^13^ In the task, the participant served as a virtual agent (the blue avatar) to play with the other two avatars (the red and the green avatar) on the screen. The participant's visual perspective was either the same (the first‐person perspective, 1PP) or different (the third‐person perspective, 3PP) with the perspective of the blue avatar, the agent they represented (Figure 2A,B). There were one practice block and 4 test blocks––the practice block composed of 36 trials with all possible conditions. The subsequent 4 test blocks were in the balanced order with 36 trials in each block. In one‐third of the trials, participants had to throw the ball—by a button press on a keyboard—to the red avatar by indicating whether the red avatar was on the left of the right side of the blue avatar (active condition). Then an animation of ball‐tossing was followed by the response. In the other trials, participants had to indicate whether the avatar on his (or her) left or right side had the ball in hand (passive condition). If they made a correct answer within the 2 s, the avatar with the ball in hand threw the ball to the blue avatar, else, the ball was thrown to the other avatar. The practice performance was monitored to ensure that participants understood the task instructions.

The view switching cost was defined as the relative IES difference between the third‐person perspective condition and first‐person perspective condition scaled by the baseline IES in the first‐person perspective condition (Equation (1)). The normalization could reduce outliers in individual analysis. The visual perspective‐taking performance defined using response times and accuracy were also analyzed and presented in the Supporting Information

(1) $$View\;switching\;cost\;=\;\frac{IES_{3PP}\;-\;IES_{1PP}}{IES_{1PP}}\;\times \;100$$

An ANOVA on the measure of view‐switching cost with tDCS condition (left, right, sham) as a within‐subject variable was performed to examine the general online tDCS effect. This analysis and the following analysis was performed using R^58^ and the “ez” package.^59^ Analysis of raw measures such as response time and accuracy with full factors was reported in the supporting information. The online neurostimulation responsiveness was defined as follows (Equations (2) and (3))

(2) $$\begin{array}{l} Online\;left\;neurostimulation\;responsiveness\\ \;\;\;=view\_switching\_cost_{sham}-view\_switching\_cost_{left} \end{array}$$

(3) $$\begin{array}{l} Online\;right\;neurostimulation\;responsiveness\\ \;\;\;=view\_switching\_cost_{sham}\;-\;view\_switching\_cost_{right} \end{array}$$

##### Behavior Tasks and Definition of Neurostimulation Responsiveness: Behavior Measure After the Stimulation (Offline)—

The imitation inhibition task was adopted as the offline behavior task utilized in previous studies.^14^, ^22^ Participants had to lift either their index or middle finger according to a color cue in each trial (see Figure 2C for an illustration of the single‐trial procedure). At the same time, a task‐irrelevant hand animation showing lifting either the same (congruent) or a different (incongruent trials) finger from that required in response to the color cue. The stimulus hand was rotated 270° anticlockwise to the participants' hand to exclude the spatial compatibility effect. Incongruent trials required participants to inhibit an imitative tendency and therefore distinguish and control motor representations evoked by the self and the other. There were 60 trials for each of the congruent and the incongruent condition, the order of which was randomized. The main effect of congruency was mainly manifested in the measure of response times (RT). The imitation cost was defined as the relative percent increase of response times in the incongruent trials compared with the congruent trials (Equation (4))

(4) $$Imitation\;cost\;=\;\frac{RT_{incongruent}\;-\;RT_{conruent}}{RT_{congruent}}\;\times \;100$$

An ANOVA on the measure of imitation cost with tDCS condition (left, right, sham) as a within‐subject variable was performed to examine the general offline tDCS effect. Analysis of raw measures such as response time and accuracy with full factors was reported in the Supporting Information. Individual stimulation responsiveness was calculated for each participants using Equations (5) and (6).

(5) $$Offline\;left\;neurostimulation\;responsiveness\;=imitation\_cost_{sham}\;-\;imitation\_cost_{left}$$

(6) $$Offline\;right\;neurostimulation\;responsiveness\;=imitation\_cost_{sham}\;-\;imitation\_cost_{right}$$

##### The Neuroimaging Session: fMRI Data Acquisition—

Participants were scanned on a 3.0‐Tesla MR system (Discovery MR750, General Electric), with an 8‐channel high‐resolution radio‐frequency head coil. Individual high‐resolution *T*_1_ images were also acquired using a 3D BRAVO EDR FAST sequence (TR = 8.15, TE = 3.17, flip angle = 12°, Matrix = 256 × 256, slice thickness = 1 mm). Functional BOLD images were acquired using the *T*_2_‐weighted echo planar imaging sequence (36 axial slices, slice thickness = 4 mm, TR = 2 s, TE = 30 ms, Matrix = 64 × 64, FOV = 220 × 220 mm). Participants completed a task‐based fMRI scanning using the false‐belief task and a resting‐state fMRI scanning. The false‐belief task composed of two conditions: (1) reading stories describing false beliefs (BELIEF) and (2) reading stories describing false photographs and maps (PHOTO). The contrast between the BELIEF and PHOTO condition can identify brain regions involved in ToM and social cognition.^60^ The task script was modified from the script obtained from the Saxelab website (https://saxelab.mit.edu/superloc.php). See supporting information for the Chinese language material used. The resting‐state fMRI comprised of 6‐minutes functional imaging during which participants were required to close their eyes and had a rest.

##### The Neuroimaging Session: Preprocessing of the False‐Belief fMRI Task—

The fMRI images were preprocessed using AFNI (Version: AFNI_18.0.00).^61^ The functional 3D volume images were corrected for slice acquisition time differences and were registered to the last volume of the second run. Then the images were spatially smoothed (FWHM = 8 mm) and each voxel time series was temporally normalized by scaling each run by its mean. In the first level GLM, the onset of BELIEF condition and PHOTO condition was convolved with a BLOCK function to represent the task regressors. Besides, head movements in the six directions were entered as covariates. The statistical map was spatially normalized by an affine transformation of the EPI data to the anatomic image followed by nonlinear registration of the anatomic data to the MNI template (MNI152_2009_template). The statistical map was resampled into the grid of 3 × 3 × 3 mm^3^ to minimize the alignment error. The beta value of the contrast between BELIEF and PHOTO was the primary interest for the following group and individual analysis. The whole‐brain group analysis was conducted on the BELIEF–PHOTO contrast value using the 3ttest^++^ program in the AFNI. A mask of mean anatomical images was used during the computation. The cluster‐size threshold was determined using nonparametric analysis (by setting the ‐Clustsim option in the 3dttest^++^ program).^62^

##### The Neuroimaging Session: Preprocessing of Resting State fMRI—

The structural *T*_1_ image was skull‐striped and spatially normalized into the MNI template (MNI152_2009_template) with the @SSwarper function. The EPI time series went through the following processing steps: de‐spiking, slice timing, head motion corrections and aligned to the MNI space at a voxel size of 3.0 × 3.0 × 3.0 mm^3^ using the output from @SSwarper, smoothing with isotropic FWHM of 6 mm, and removal of noise using ANATICOR.^63^ The residual time course after regressing out noises was used for seed‐based functional connectivity analysis.

##### The Neuroimaging Session: Definition of the TPJ—

We constructed a broadly defined TPJ mask as the procedure described in previous literature.^33^ Specifically, The TPJ mask comprised the supramarginal gyrus, posterior superior temporal gyrus, posterior superior temporal sulcus, and angular gyrus from the Harvard–Oxford probabilistic brain atlas embedded in the FSL.^64^ The anterior border (at *y* = 16 mm) and the ventral border (at *z* = 0 mm) was used to preclude brain regions that were not likely to be the TPJ. Then, the TPJ mask was further divided into three parts using Yeo's seven network atlas.^65^ The Yeo mask in the nonlinear MNI152 space was extracted from https://surfer.nmr.mgh.harvard.edu/fswiki/CorticalParcellation_Yeo2011.The 3 subdivision of TPJ were an IPL part associated with the frontoparietal network (FPN), a posterior part (TPJp) associated with the default mode network (DMN), and an anterior part (TPJa) associated with the cingulo‐opercular network (CON). To validate the division of TPJ, we performed a seed‐based functional connectivity analysis. The mean time course within IPL, TPJa, TPJp were averaged, and the Fisher‐z transformed correlation map was computed using the AFNI command 3dTcorr1D. The mean IPL, TPJa, and TPJp network was then calculated to validate the division of TPJ into three network components. The left IPL was used as the seed to perform a seed‐based functional connectivity analysis to get the specific left IPL network.

##### The Neuroimaging Session: Calculation of Lateralization Index (LI)—

We calculate the lateralization index (LI) of the IPL, TPJa, and TPJp mask using the LI_toolbox.^32^ The LI_toolbox extracted bootstrapped samples in the left and right ROIs at a range of thresholds. Only the central 50% of LI combinations of each individual were retained to avoid the influence of statistical outliers. A weighted mean LI was then calculated. Higher thresholds received higher weights in the resulting index to minimize the influence of arbitrarily chosen thresholds. We flip the sign of the LI index from the LI_toolbox by multiplying −1 so that a positive value indicating rightward lateralization.

##### Analyze the Association between LI and Neurostimulation Responsiveness: Compare the Contribution of the 3 Division of TPJ—

We use linear regression models using R^58^ to quantify the independent contribution of IPL, TPJa, TPJp in predicting neurostimulation responsiveness. See supporting information (Tables S7, S8, S9, and S10, Supporting Information) for the model specification.

##### Analyze the Association between LI and Neurostimulation Responsiveness: Compare the Contribution of Left and Right Activations—

The average activation in the left and the right side of the ROI (such as IPL_left and IPL_right) was calculated by only including voxels having positive belief‐photo contrast. The following linear regression models were fitted to quantify the independent contribution of left and right IPL in predicting neurostimulation responsiveness. See supporting information (Tables S11, S12, S13, and S14, Supporting Information) for the statistical summary for each model.

##### Analyze the Association between LI and Neurostimulation Responsiveness: The Positive Responders Versus the Negative Responders—

First, we performed a hierarchical cluster analysis according to the four measures of neurostimulation responsiveness: online left, online right, offline left, and offline right neurostimulation responsiveness using R (R Core Team, 2018). Second, we examined the profile of positive responders and negative responders by using a *t*‐test on the four kinds of neurostimulation responsiveness. Third, we examined the group difference in the measure of LI. Fourth, we tested whether the LI difference originated from a stronger left TPJ activation or a weaker right TPJ activation. Finally, we tested the group difference in social traits. Besides, the association between LI, social traits, and neurostimulation responsiveness was also examined.

We also tested the feasibility of the prestimulation model using simple measures such as LI and social traits using logistic regression (R Core Team, 2018). A model comparison approach was used to compare the importance of the psychological and neural predictors and select the optimal model. The detail of receiver operator curve analysis and leave‐one‐out‐cross‐validation was provided in the supporting information. See Supporting Information: 6. A prestimulation screening model using simple measures for detail information.

##### Analyze the Association Between LI and Neurostimulation Responsiveness: Mediation Analysis Examining the Role of IPL‐meSFG Connectivity—

A seed‐based analysis was performed using the bilateral IPL as the seed and compared the IPL network strength between positive responders and negative responders using the whole‐brain *t*‐test. The individual IPL‐meSFG connectivity was extracted. Then, the mediation analysis was performed to examine whether the IPL‐meSFG connectivity mediated the prediction of neurostimulation responsiveness by lateralization and social trait. The “psych” package of R was used to fit the mediation model, and the statistical inference of the indirect effect was determined using the bootstrapping procedure.

## Conflict of Interest

The authors declare no conflict of interest.

## Supporting information

Supporting Information
