# Supplementary material for: Neural and Psychological Predictors of Cognitive Enhancement and Impairment from Neurostimulation
Source: Adv Sci (Weinh). 2020 Jan 21;7(4):1902863. doi: 10.1002/advs.201902863 (PMC7029648; doi:10.1002/advs.201902863)
Supplement: Supplementary file 1 — Supporting Information [file ADVS-7-1902863-s001.pdf]

Copyright WILEY-VCH Verlag GmbH & Co. KGaA, 69469 Weinheim, Germany, 2020.

ADVANCED SCIENCE
Open Access

Supporting Information

for *Adv. Sci.,* DOI: 10.1002/advs.201902863

Neural and Psychological Predictors of Cognitive Enhancement and Impairment from Neurostimulation

Li-Zhuang Yang, Wei Zhang, Wenjuan Wang, Zhiyu Yang, Hongzhi Wang, Zhi-De Deng, Chuanfu Li, Bensheng Qiu, Da-Ren Zhang, Roi Cohen Kadosh, Hai Li, and Xiaochu Zhang**

Supporting Information

Neural and psychological predictors of cognitive enhancement and impairment due to neurostimulation

Li-Zhuang Yang[1,2,3], Wei Zhang[3], Wenjuan Wang[3], Zhiyu Yang[3], Hongzhi Wang[2], Zhi-De Deng[4], Chuanfu Li[5], Bensheng Qiu[6], Da-Ren Zhang[3], Roi Cohen Kadosh[7], Hai Li[1,2], Xiaochu Zhang[3,6,8,9*]*

Contents

1. Sample size calculation
2. Complementary analysis of the behavior data in the tDCS experiment
2.1 Online task: the visual perspective taking task
2.2 Offline task: the imitation inhibition task
3. Compare the contribution of LI of 3 TPJ divisions
4. Examine the contribution of left and right TPJ_IPL
5. The profile of cognitive and negative responders
6. A pre-stimulation screening model using simple measures
7. The Chinese materials of the false-belief task
8. A discussion on the feasibility of the pre-stimulation screening approach

1. Sample Size Calculation

The main purpose of the present study was to investigate the association between measures of hemispheric communication and stimulation responsiveness. Assuming a correlation coefficient of medium effect size (r = 0.4) and a power of 80%, a sample of 46 subjects were needed. The calculation was performed using the 'pwd' package in R environment [1].

Fifty-four subjects were recruited in the study. However, the actual final sample size with both fMRI and tDCS data available was 45 because of drop-outs (seven drop-outs in tDCS session and 1 drop-out in the fMRI session) and data quality issues (one participant with excessive head motion).

2. Complementary analysis of the behavior data in the tDCS experiment

2.1. Online task: the visual perspective taking task

We conducted full factorial ANOVAs with 3 within-subject variables: agent (active vs. passive), perspective (1st Perspective vs. 3rd Perspective) and tDCS condition (left stimulation, sham stimulation, right stimulation) on raw measures of Inverse Efficiency Score (*IES*) (**Table S1**), response times (*RTs*) (**Table S2**), and accuracy (**Table S3**), separately. All following analysis was performed within R environment [2] using the "ez" package [3].

2.1.1. A full factorial ANOVA analysis on IES

Table S1. Summary table of the 3-factor omnibus ANOVA on *IES*

Effect	*df1*	*df2*	*F*	*p*	η^2
tDCS condition	2	88	2.934	.058	.013
Perspective	1	44	156.388	< .001	.423
Agent	1	44	19.662	< .001	.017
tDCS : Perspective	2	88	2.500	.088	.002
tDCS : Agent	2	88	0.507	.604	.000
Perspective : Agent	1	44	27.415	< .001	.017
tDCS : Perspective : Agent	2	88	1.362	.261	.000

2.1.2. A full factorial ANOVA analysis on RTs

Table S2. Summary table of the 3-factor omnibus ANOVA on *RTs*

Effect	*df1*	*df2*	*F*	*p*	η^2
tDCS condition	2	88	2.829	.064	.015
Perspective	1	44	483.890	< .001	.604
Agent	1	44	46.014	< .001	.016
tDCS : Perspective	2	88	.084	.919	.000
tDCS : Agent	2	88	0.562	.572	.000
Perspective : Agent	1	44	77.124	< .001	.002
tDCS : Perspective : Agent	2	88	1.225	.298	.000

2.1.3. A full factorial ANOVA analysis on Accuracy

Table S3. Summary table of the 3-factor omnibus ANOVA on *Accuracy*

Effect	*df1*	*df2*	*F*	*p*	η^2
tDCS condition	2	88	2.044	.140	.008
Perspective	1	44	45.211	< .001	.179
Agent	1	44	4.838	.003	.007
tDCS : Perspective	2	88	1.816	.169	.002
tDCS : Agent	2	88	0.621	.540	.000
Perspective : Agent	1	44	6.476	.015	.004
tDCS : Perspective : Agent	2	88	.316	.730	.000

2.2. Offline task: the imitation inhibition task

We conducted full factorial ANOVAs with 2 within-subject variables: congruency (congruent vs. incongruent), and tDCS condition (left stimulation, sham stimulation, right stimulation) on raw measures of Inverse Efficiency Score (IES) (**Table S4**), response times (RTs) (**Table S5**), and accuracy (**Table S6**), separately.

2.2.1. A full factorial ANOVA analysis on IES

Table S4. Summary table of the 2-factor omnibus ANOVA on *IES of the imitation task*

Effect	*df1*	*df2*	*F*	*p*	η^2
tDCS condition	2	88	.310	.073	< .001
Congruency	1	44	98.987	< .001	.140
tDCS : Congruency	2	88	1.701	.189	< .001

2.2.2. A full factorial ANOVA analysis on response times

Table S5. Summary table of the 2-factor omnibus ANOVA on *RT of the imitation task*

Effect	*df1*	*df2*	*F*	*p*	η^2
tDCS condition	2	88	.076	.927	< .001
Congruency	1	44	132.711	< .001	.087
tDCS : Congruency	2	88	2.982	.056	.001

2.2.3. A full factorial ANOVA analysis on accuracy

Table S6. Summary table of the 2-factor omnibus ANOVA on *accuracy of the imitation task*

Effect	*df1*	*df2*	*F*	*p*	η^2
tDCS condition	2	88	.661	.519	.003
Congruency	1	44	34.579	< .001	.138
tDCS : Congruency	2	88	.018	.982	<.001

3. Compare the contribution of LI of 3 TPJ divisions

To compare the contribution of LI of the three sub-divisions of TPJ, we fitted 4 regression models with the three LI as predicting variables to explain online left neurostimulation responsiveness, online right neurostimulation responsiveness, offline left neurostimulation responsiveness and offline right neurostimulation responsiveness, respectively (See **Table S5**, **S6**, **S7**, and **S8** for detail statistical summary table for each regression model). We found a consistent contribution of the LI of the IPL part. Specifically, the LI is generally negatively associated with the neurostimulation responsiveness suggesting an important role of left TPJ in promoting positive neurostimulation responsiveness (**Figure S1**).

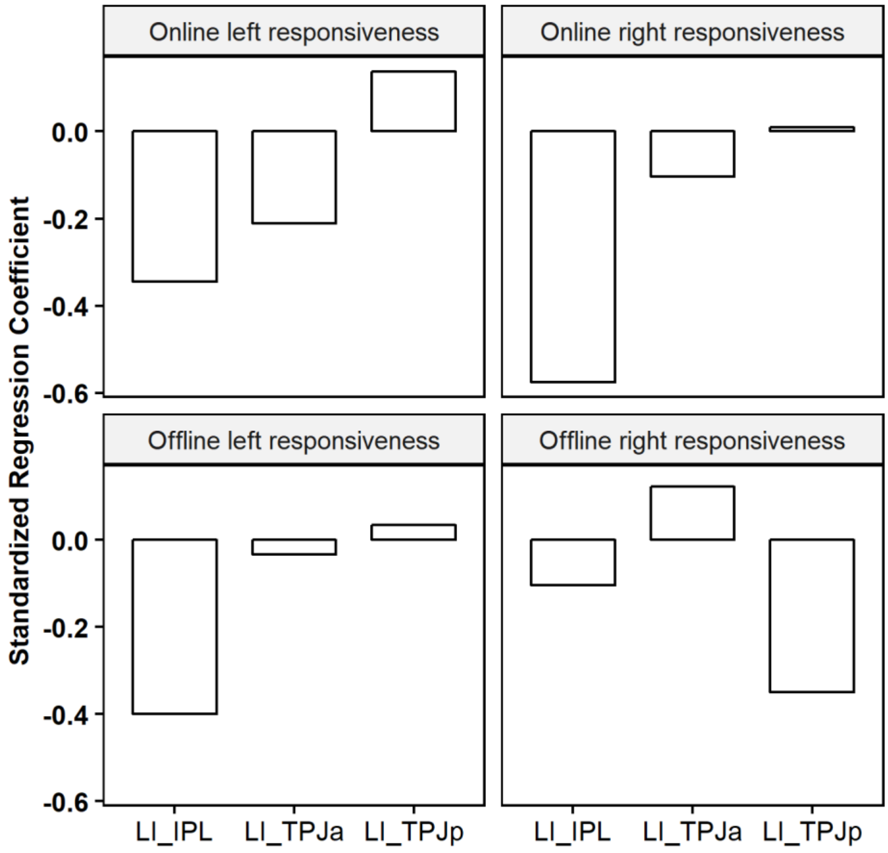

Figure S1. The standard regression coefficient of LI_IPL, LI_TPJa, LI_TPJp in predicting online left neurostimulation responsiveness, online right neurostimulation responsiveness, offline left neurostimulation responsiveness and offline right neurostimulation responsiveness. Note: this plot was merely used to illustrate the opposite contribution of left and right IPL in predicting neurostimulation responsiveness.

Model 1: Online left stimulation responsiveness ~ LI_IPL + LI_TPJa + LI_TPJp

Table S7. Summary of model 1.

Variable	Standardized regression coefficients	Standard Error	t	p
LI_IPL	-.345	.158	-2.181	.035
LI_TPJa	-.211	-.178	-1.183	.244
LI_TPJp	.137	.191	.715	.479
R^2		.131		
Adjusted R^2		.068		
F		2.068		.119

Model 2: Online right stimulation responsiveness ~ LI_IPL + LI_TPJa + LI_TPJp

Table S8. Summary of model 2.

Variable	Standardized regression coefficients	Standard Error	t	p
LI_IPL	-.575	.124	-4.205	.0001
LI_TPJa	-.104	-.154	-.675	.503
LI_TPJp	.957	.165	.058	.954
R^2		.351		
Adjusted R^2		.303		
F		7.384		.0004

Model 3: Offline left stimulation responsiveness ~ LI_IPL + LI_TPJa + LI_TPJp

Table S9. Summary of model 3.

Variable	Standardized regression coefficients	Standard Error	t	p
LI_IPL	-.400	.156	-2.563	.014
LI_TPJa	-.034	-.176	-.193	.848
LI_TPJp	.034	.189	.181	.857
R^2		.154		
Adjusted R^2		.092		
F		2.492		.074

Model 4: Offline right stimulation responsiveness ~ LI_IPL + LI_TPJa + LI_TPJp

Table S10. Summary of model 4.

Variable	Standardized regression coefficients	Standard Error	t	p
LI_IPL	-.104	.159	-.655	.516
LI_TPJa	-.123	-.179	-.685	.497
LI_TPJp	-.350	.920	-1.824	.075
R^2		.124		
Adjusted R^2		.060		
F		1.932		.139

4. Examine the contribution of left and right TPJ_IPL

To examine the specific contribution of left and right TPJ, especially the IPL, in predicting the neurostimulation responsiveness, we fitted 4 regression models with left and right activations

of IPL as predicting variables to explain the online left neurostimulation responsiveness, the online right neurostimulation responsiveness, the offline left neurostimulation responsiveness, and the offline right neurostimulation responsiveness, respectively.

Model 5: Online left stimulation responsiveness ~ IPL_left + IPL_Right

Table S11. Summary of model 5.

Variable	Standardized regression coefficients	Standard Error	t	p
IPL_Left	.247	.182	1.355	.183
IPL_Right	-.050	-.182	-.272	.787
R^2		.050		
Adjusted R^2		.004		
F		1.097		.343

Model 6: Online right stimulation responsiveness ~ IPL_left + IPL_Right

Table S12. Summary of model 6.

Variable	Standardized regression coefficients	Standard Error	t	p
IPL_Left	.504	.170	2.962	.005
IPL_Right	-.253	.170	-1.487	.145
R^2		.174		
Adjusted R^2		.134		
F		4.413		.018

Model 7: Offline left stimulation responsiveness ~ IPL_left + IPL_Right

Table S13. Summary of model 7.

Variable	Standardized regression coefficients	Standard Error	t	p
IPL_Left	.469	.171	2.736	.009
IPL_Right	-.367	.171	-2.142	.038
R^2		.160		
Adjusted R^2		.120		
F		4.004		.026

Model 8: Offline right stimulation responsiveness ~ IPL_left + IPL_Right

Table S14. Summary of model 8.

Variable	Standardized regression coefficients	Standard Error	t	p
IPL_Left	.371	.178	2.084	.043
IPL_Right	-.255	.178	-1.431	.160
R^2		.096		
Adjusted R^2		.053		
F		2.219		.121

5. The profile of cognitive and negative responders

The positive responders showed significant positive neurostimulation responsiveness indicating a gain of performance after active stimulation [Online left: t = 3.743, df = 16, p = .002, Cohen's d = .908; Online right: t = 4.318, df = 16, p < .001, Cohen's d = 1.048; Offline left: t = 2.097, df = 16, p = .052, Cohen's d = .508; Offline right: t = 2.389, df = 16, p = .029, Cohen's d = .579]. While the negative responders showed significant negative neurostimulation responsiveness indicating a loss of performance after active stimulation [Online left: t = -2.877, df = 27, p = .008, Cohen's d = .544; Online right: t = -2.093, df = 27, p = .046, Cohen's d = .395; Offline left: t = -5.701, df = 27, p < .001, Cohen's d = 1.078; Offline right: t = -4.419, df = 27, p < .001, Cohen's d = .835]. See **Figure S2** for an illustration.

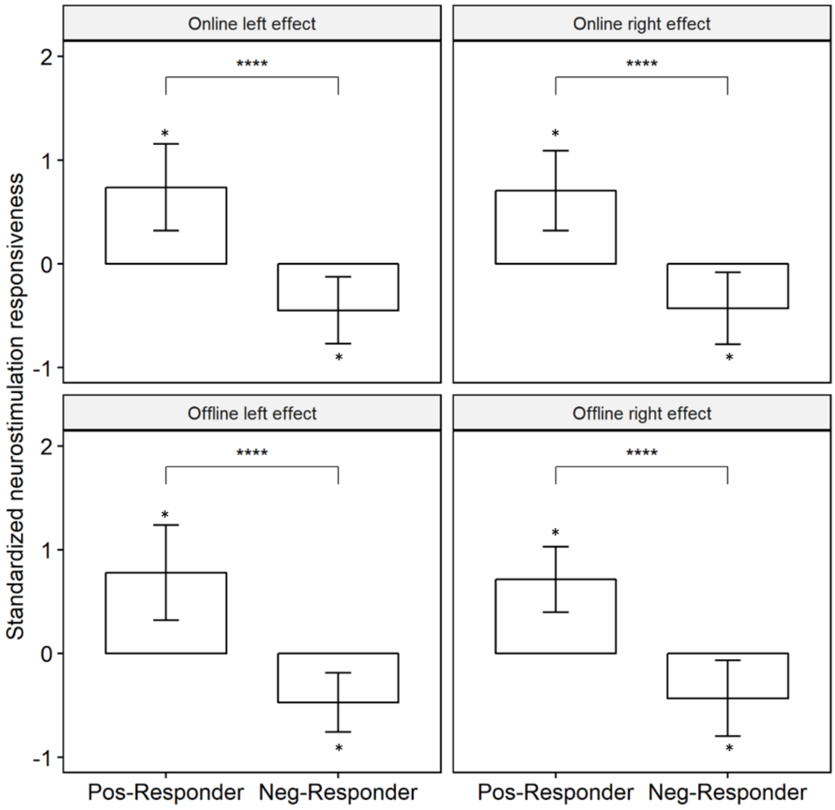

Figure S2. The neurostimulation responsiveness profile of positive responder and negative responder, which was classified using hierarchical cluster analysis. The error bar represents the 95% confidence interval of the mean. **** indicates a significance level of $p < .0001$.

The positive responders had a lower level of rightward lateralization of IPL than the negative responders [t = 2.953, df = 30.211, p = .006, Cohen's d = .941]. However, there was no difference between the positive responders and the negative responders on lateralization of TPJa [t = .071, df = 26.938, p = .944, Cohen's d = .023] and TPJp [t = 1.388, df = 35.966, p = .174, Cohen's d = .419]. See **Figure S3** for a visual illustration.

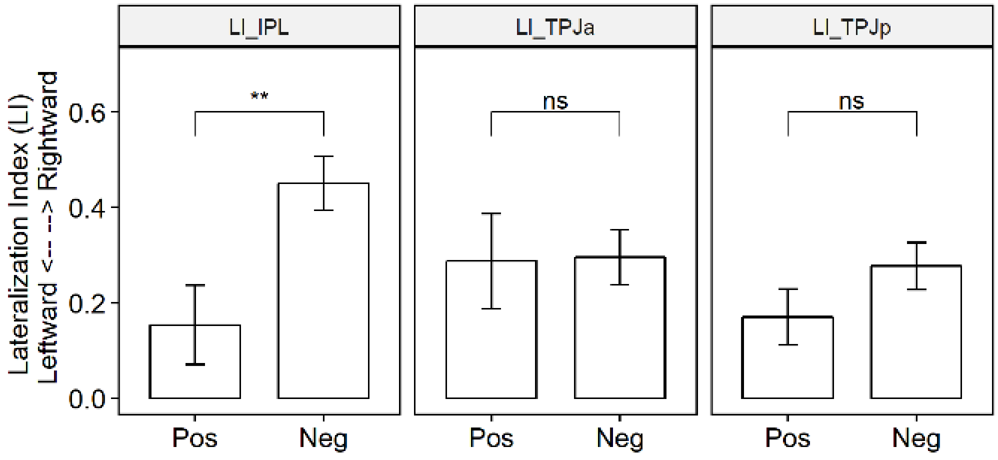

Figure S3. The difference of LI between the positive responders and the negative responders. Pos: Positive responders. Neg: Negative responders.

Moreover, the positive responders showed a stronger left IPL activation than the negative responders [t = 2.314, df = 30.027, p = .028, Cohen's d = .739]. However, the two group were not different from each other on the right IPL activation [t = .005, df = 34.718, p = .996, Cohen's d = .002] (**Figure S4**).

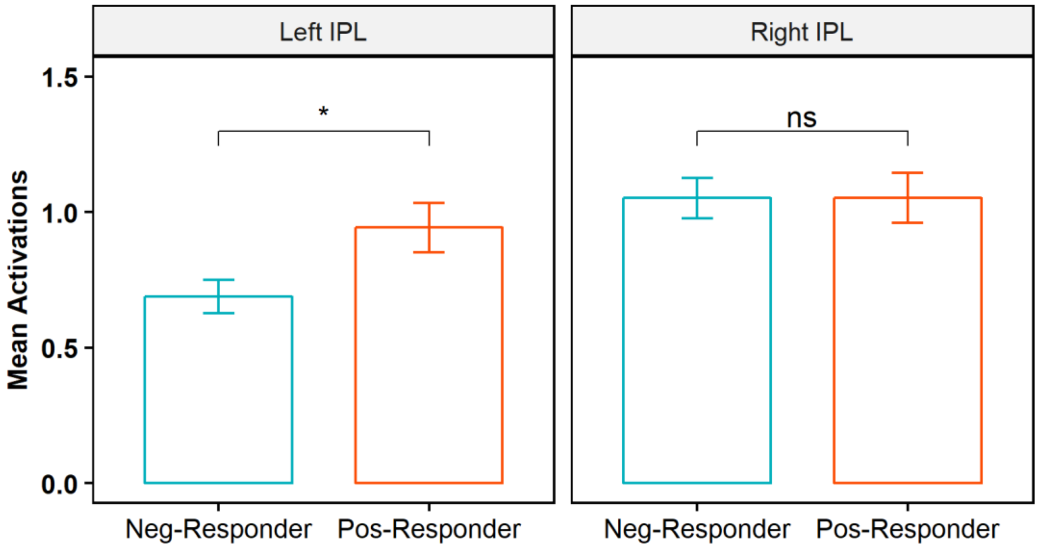

Figure S4. Positive and negative responders' mean IPL activations in the false-belief fMRI task. * indicating a significance level of p < .05. Pos-Responder: Positive

responder. Neg-Reponder: Negative responder.

Interestingly, the positive responders and the negative responders also differed on social traits. See Table S15 for detail statistics.

Table S15. Summary of model 8.

	Social Phobia Scale	Autism Quotient
positive responders (*n* = 17)	48.118 ± 16.959	23.824 ± 5.423
negative responders (n = 28)	34.630 ± 19.156	20.704 ± 5.703
t	2.442	1.821
p	.019	.077
Cohen's D	.735	.557

6. A pre-stimulation screening model using simple measures

We tested the feasibility of the pre-stimulation model using simple measures such as LI and social traits using logistic regression (R Core Team, 2018). The receiver operator curve analysis was performed using the “pROC” package [4] and the leave one out cross validation was performed using the “Caret” package in the R environment.

We calculated a composite LI by averaging the three LI indexes, namely LI_IPL, LI_TPJa, and LI_TPJp to indicate the general lateralization of TPJ and a social trait score by averaging the score of the social phobia scale and the Autism Quotient to represent participants’ social traits. Firstly, we fitted a baseline logistic model using only the composite social trait score (logistic model 1: responder ~ social). Secondly, we fitted the model with the composite LI and the composite social traits (logistic model 2: responder ~ social + LI). Thirdly, we

examined whether there was an interaction between LI and social trait by modeling an interaction term (logistic model 3: responder ~ social + LI + social × LI). The unique contribution of LI was verified by model comparison using the χ^2 test. LI improved the mode fit significantly (logistic model 2 vs. logistic model 1: χ^2 = 6.554, p = .010). And, there was no significant interaction between LI and social trait (logistic model 3 vs. logistic model 2: χ^2 = 3.623, p = .057). We also evaluate the performance of those three models using Leave-One-Out-Cross-Validation (LOOCV). The package "Caret" in R [5] was used to compute the accuracy and kappa coefficient. The logistic model 2, including only the main effect of social trait score and LI, achieved the same classifying accuracy as the full model (logistic model 3) and highest Kappa coefficient (**Table S16**).

Table S16. The accuracy and kappa coefficient using Leave-One-Out-Cross-Validation

Model Specification	Classifying Accuracy	Kappa Coefficient
Logistic model 1: *Responder ~ social*	.591	.055
Logistic model 2: *Responder ~ social + LI*	**.636**	**.198**
Logistic model 3: *Responder ~ social + LI + social× LI*	.636	.179

7. The Chinese materials of the false-belief task

The Chinese items of the false belief task is attached.

False-belief stories:

(1) 校园舞会当日的清晨，小红将她的高跟鞋放在连衣裙下后出门购物。当天中午，她的姐姐借用了那双鞋并在事后将鞋放在小红床下。

(2) 小红离开的时候，小明正在沙滩上熟睡。几分钟后小明被海浪惊醒，发现小红已经离开了，他决定去游个泳。

(3) 小明告诉小红他丢了钥匙，他们一起在屋子里寻找，但没有找到。小红出了门到车里搜寻，这时小明发现他的钥匙落在了在沙发后面。

(4) 一个清洁工被委托清洁某公司大楼的玻璃。但工作进行一半后清洁工具中途损坏导致他无法完成清洁。第二天早晨，CEO 来到了公司。

(5) 小红和小明提前预定了回国的客舱，但负责人忘记记下他们的预约，而另外两位乘客率先进入了客舱。

(6) 小明为自己将在周五提交的论文选择了一个尚无定论的话题。周四的新闻表明那个话题已经得到了解释，但是小明并没有看到这个消息。

(7) 放学后老师让小红叫小明去办公室，小红回到教室寻找小明，别人却告诉她小明在半个小时前就已经回家去了。

(8) 小明的父母今晚将在餐厅庆祝结婚纪念日，他们给小明留了字条，但在他们离开后字条票飘到沙发下面了。小明回到家发现家中没有人。

(9) 小红在小明离开的时候弄坏了他的模型玩具，她迅速从店里买了一个一模一样的放回原来的位置。小明回来后看到了模型玩具。

(10) 小明在学校弄坏了雨伞，妈妈趁小明睡觉的时候把雨伞修好放在了小明的书包旁边。小明第二天起床后背着书包去上学。

(11) 小明在学校嘲笑小红不会跳绳，小红回家后在父亲的指导下学会了跳绳。第二天一早，小明依然嘲笑小红不会跳绳。

(12) 小红把买来的点心放在冰箱里准备晚餐后吃，就在她准备晚餐的时候，小明回到家把点心吃掉了。晚餐后，小红打开冰箱。

(13) 小明知道小红从今天开始要出差，来到单位看到小红的座位是空的。实际上小红发烧了无法工作，她打电话退掉了机票。

(14) 小明整理行李的时候将充电器放进了背包，当天晚上小红又把它拿进了自己的房间给手机充电。第二天一早小明就背着包离开了。

False-photo stories

(1) 在绘制这幅肖像的时候，模特是一位留着棕色短发、未蓄胡须的年轻男性。而现在他留起了长发，而且和胡须一样，都成了灰色。

(2) 在中学时期的照片里，这个女孩穿着白色的外套。后来，她不小心将这件外套和一双红色的袜子一起洗，外套变成了粉红色。

(3) 很久以前，一个探险家绘制了某岛屿的地图。在那之后，水位不断上涨，那个岛屿已经只剩极小的一部分暴露在水面上。

(4) 照这张像的时候，这栋建筑只有一层楼高。而在那之后，翻修者又为它增加了一层楼以及一个车库。

(5) 三个月前，加勒比岛上的一座火山爆发。现在岛上只剩下荒芜的火山岩。卫星照片显示了火山爆发前岛上的景象。

(6) 自礼堂落成的那天起，这棵橡木树就立在它的门前。去年这棵树倒下了，礼堂门前的景物被换成了一座大理石喷泉。

(7) 海洋博物馆中展示着关于泰坦周边岛屿的老地图。后来在海水的不断侵蚀下，只剩下了三个最大的主要岛屿。

(8) 作者以八十年代社会为背景创作了这部小说，那时移动通讯设备并不像当下这样普及，书信依然是人们联络的主要方式。.

(9) 屋主的父亲买下这栋房子时在院子里栽了一棵苹果树，但屋主在翻修房屋的同时推到了苹果树，改种了一棵梨树。

(10) 最早的笔记本电脑足有六部苹果手机那么厚，然而随着科技的发展，如今很多便携式电脑只有一份报纸那么薄。

(11) 第一版的教科书是繁体字写成的。在之后的几十年间，教科书的内容经过了多次的修订，文字也在二十年前由繁体改成了简体。

(12) 很久以前这里还只是一座萧条的小镇，旅行商人的来到改变了小镇的经济状况。通过出口当地特有的商品，小镇如今已繁荣了起来。

(13) 海盗将宝藏埋在了太平洋的某个岛屿上并绘制了藏宝图，但由于板块运动如今那座岛屿的位置已经发生了巨大的变化。

(14) 主人少年时得到的这辆自行车曾经是最新的款式，如今它当年光鲜的表面变得斑斑驳驳，链条上挂满了铁锈，刹车也已经失灵。

8. A discussion on the feasibility of the pre-stimulation screening approach

Firstly, the procedure for constructing a pre-stimulation model in the present study is replicable. We only used the functional lateralization index and the social-related trait to predict responsiveness to tDCS. The measurement of lateralization is intuitive and calculated using the validated open-sourced toolbox. Besides, the measure of social-related traits indicates the necessity to receive treatment. The construction of the prediction model all based on the open-source package in the R environment. We think clinical practitioners can grasp the methodology without effort and construct models using their local samples.

Secondly, the association between lateralization index and responsiveness to tDCS, revealed by our study, is generalizable. In the old manuscript, we only supported the association

between LI and responsiveness using the visual perspective-taking task at the online phase. In the new manuscript, we add analysis on the offline task, the imitation inhibition task. We demonstrate the association between LI and responsiveness can generalize to the offline phase, which uses an independent behavioral measurement, namely the imitation inhibition task. The evidence of generalization supports the effectiveness of our modeling strategy.

Reference

[1] S. Champely, 2018.

[2] R Core Team, R Foundation for Statistical Computing, Vienna, Austria 2018.

[3] M. A. Lawrence, 2016.

[4] X. Robin, N. Turck, A. Hainard, N. Tiberti, F. Lisacek, J. C. Sanchez, M. Muller, Bmc Bioinformatics 2011, 12.

[5] M. Kuhn, 2018.
